# TIPS: a novel pathway-guided joint model for transcriptome-wide association studies

**DOI:** 10.1093/bib/bbae587

**Published:** 2024-11-16

**Authors:** Neng Wang, Zhenyao Ye, Tianzhou Ma

**Affiliations:** Department of Mathematics, University of Maryland, College Park, MD 20742, United States; Department of Epidemiology and Biostatistics, University of Maryland, College Park, MD 20742, United States; Department of Epidemiology and Public Health, University of Maryland, Baltimore, MD 21201, United States; Department of Epidemiology and Biostatistics, University of Maryland, College Park, MD 20742, United States

**Keywords:** transcriptome-wide association studies, biological pathways, sparse group lasso, EM algorithm

## Abstract

In the past two decades, genome-wide association studies (GWAS) have pinpointed numerous SNPs linked to human diseases and traits, yet many of these SNPs are in non-coding regions and hard to interpret. Transcriptome-wide association studies (TWAS) integrate GWAS and expression reference panels to identify the associations at gene level with tissue specificity, potentially improving the interpretability. However, the list of individual genes identified from univariate TWAS contains little unifying biological theme, leaving the underlying mechanisms largely elusive. In this paper, we propose a novel multivariate TWAS method that Incorporates Pathway or gene Set information, namely TIPS, to identify genes and pathways most associated with complex polygenic traits. We jointly modeled the imputation and association steps in TWAS, incorporated a sparse group lasso penalty in the model to induce selection at both gene and pathway levels and developed an expectation-maximization algorithm to estimate the parameters for the penalized likelihood. We applied our method to three different complex traits: systolic and diastolic blood pressure, as well as a brain aging biomarker white matter brain age gap in UK Biobank and identified critical biologically relevant pathways and genes associated with these traits. These pathways cannot be detected by traditional univariate TWAS + pathway enrichment analysis approach, showing the power of our model. We also conducted comprehensive simulations with varying heritability levels and genetic architectures and showed our method outperformed other established TWAS methods in feature selection, statistical power, and prediction. The R package that implements TIPS is available at https://github.com/nwang123/TIPS.

## Introduction

During the past two decades, large-scale genome-wide association studies (GWAS) have identified thousands of genetic variants (typically SNPs) that influence risk of numerous human traits and diseases [1]. However, the path from GWAS to biological understanding remains unclear is still not clear as an SNP–trait association cannot be directly used to infer the target gene or the mechanism whereby the variant is associated with phenotypic differences. Underscoring a refined focus on the transcriptome’s role in understanding genetic influences on complex traits, recent developments in genetic epidemiology have shifted the paradigm from GWAS to transcriptome-wide association studies (TWAS), which leverage expression reference panels (expression quantitative trait loci (eQTL) cohorts like GTEx [2], which has both expression and genotype data available) to discover gene–trait associations from GWAS datasets [3]. Since the birth of the first TWAS method [4], many statistical methods have been developed to perform TWAS [5–9]. These methods are typically implemented in two stages: an imputation stage that utilizes the reference dataset to ‘impute’ gene expression for each individual and the association analysis stage that correlates each individual’s ‘imputed’ gene expression with their phenotype. Alternatively, joint models of TWAS that combine the two stages together are also being developed and emerge as a more holistic approach to account for the imputation uncertainty [10, 11]. Such TWAS methods typically use likelihood-based inference and have been shown to be more powerful and efficient than the two-stage methods.

Most complex traits and diseases are polygenic by nature, with their heritability depending on a large number of genes across the genome. Like GWAS, the standard TWAS is essentially a univariate analysis where each gene is tested at a time. However, genes do not function in isolation, rather they act cooperatively in groups (e.g., biological pathways) to perform biological functions. Such a univariate analysis provides the contribution of each single gene to the trait with little unifying biological themes. In addition, univariate analysis ignores the complex correlation between genes making it difficult to identify the most critical and potentially causal genes of a trait. Development of multivariate TWAS is still in its infancy and existing methods mainly target at specific risk regions and are strictly two stage methods only [12–14]. To the best of our knowledge, no methods have ever considered the gene–gene correlation, gene sets with similar function on a broad transcriptome-wide scale in prioritizing the most critical genes and pathways that contribute to a polygenic trait while in the same time accounting for the imputation uncertainty in TWAS.

**Figure 1:**
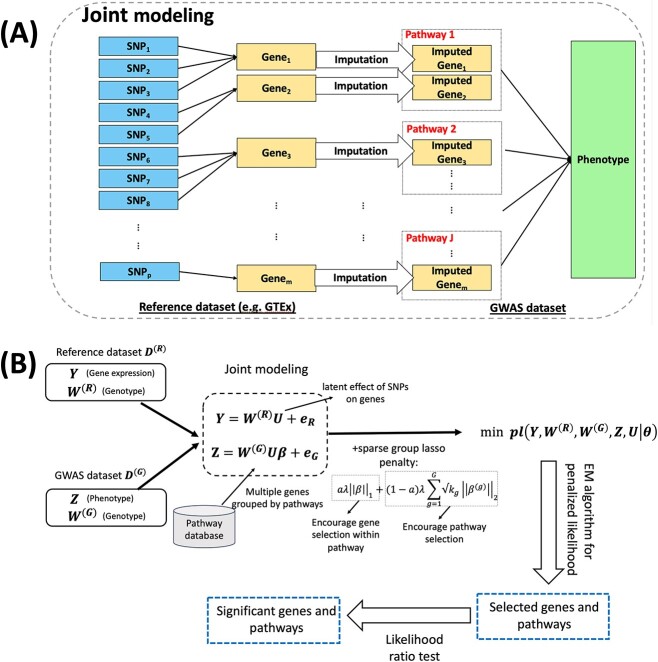
(A) Conceptual framework to motivate the TIPS model. (B) Flowchart of the TIPS method.

To fill this gap, we developed a novel multivariate TWAS method that Incorporates Pathway or gene Set information, namely TIPS, and utilized a sparse group lasso penalty to select the most important genes and pathways that contribute to a polygenic trait. Figure 1(A) shows a conceptual framework that motivates our model. As in most TWAS methods, we assume each gene can be regulated by multiple SNPs (i.e. eQTLs) in the reference dataset. Unlike existing univariate TWAS methods that perform association analysis for one gene at a time, TIPS examines multiple genes potentially grouped in biological pathways concurrently, capturing their collective impact on the trait. Such a multivariate pathway guided approach is essential for a comprehensive understanding of the genetic architecture of a complex polygenic trait and revealing the biological processes underlying the trait. Furthermore, TIPS integrates the imputation and association analysis stages together to enhance the efficiency of the model and develops an Expectation-Maximization (EM) algorithm for penalized likelihood to estimate the parameters and select both individual- (genes) and group-level (pathways) features. In addition, we employed a data splitting scheme to perform post-selection inference at both gene and pathway levels using likelihood ratio tests (LRTs). We showed the advantage of TIPS over existing TWAS methods in extensive simulations and via TWAS analysis on three different complex traits: systolic blood pressure (SBP), diastolic blood pressure (DBP), and a brain aging biomarker white matter (WM) Brain Age Gap (BAG), in UK Biobank (UKB).

The paper is organized as follows. In Section 2, we introduced the TIPS model, the penalized likelihood, the EM algorithm to estimate the parameters, and LRT for post-selection inference. In Section 3, we applied our method to GWAS data in UKB to identify the genes and pathways contributing to the variations of three phenotypes (SBP, DBP and WM BAG). In Section 4, we conducted various simulations to show the advantages of our method over other TWAS methods in feature selection, statistical power and prediction. In Section 5, we provided discussion on the potential extension of our method in future studies.

## Method

### The TIPS model

Figure 1(B) shows a flowchart of the TIPS method. Denote the reference dataset (e.g., GTEx) by $\mathbf{D^{(R)}=\{Y; W^{(R)}\}}$, where $\mathbf{Y}$ ($n_{1} \times m$) corresponds to the gene expression data matrix and $\mathbf{W^{(R)}}$ ($n_{1} \times p$) the genotype data matrix in the reference dataset. $n_{1}$ refers to the sample size in reference dataset, $m$ indicates the number of genes potentially predictive of phenotype of interest, and $p$ represents the total number of genetic variants that potentially regulate these genes. Denote the GWAS dataset by $\mathbf{D^{(G)}=\{Z; W^{(G)}\}}$, where $\mathbf{Z}$ is an ($n_{2} \times 1$) vector of the phenotype of interest and $\mathbf{W^{(G)}}$ ($n_{2} \times p$) corresponds to the genotype data matrix from GWAS. $n_{2}$ refers to the sample size in the GWAS dataset.

TIPS jointly models $\mathbf{Y}$, $\mathbf{W^{(R)}}$, $\mathbf{W^{(G)}}$ and $\mathbf{Z}$ together and performs imputation and association analysis simultaneously using the following model consisting of two regression equations:


(1) \begin{align*}& \begin{split} \mathbf{Y} & = \mathbf{W^{(R)} U} + \mathbf{e_{R}}; \\ \mathbf{Z} & = \mathbf{W^{(G)} U}\mathbf{\beta} + \mathbf{e_{G}}, \end{split}\end{align*}

where $\mathbf{U}$ is a $(p \times m)$ matrix representing the genetic effects on the gene expression. In general, $\mathbf{U}$ matrix can have arbitrary elements to represent the complex SNP–gene regulatory relationship. If we only consider the cis regulatory effect on gene expression (i.e. cis-eQTL) and assume no common regulatory variants shared among genes, then $\mathbf{U}$ matrix can be simplified to a sparse block matrix with off-block elements being zero. Following [15], we further assume a multivariate normal distribution as a prior on $\mathbf{U}$: $\mathbf{U} \sim \mathcal{MN}_{p\times m}(0_{p\times m}, I_{p}, \Sigma _{u} )$, where $I_{p}$ is the $(p\times p)$ identity matrix and $\Sigma _{u}$ is an $(m\times m)$ diagonal matrix with $j$th ($1\le j \le m$) diagonal element being $\sigma _{u_{j}}^{2}$, by assuming SNP–gene regulatory effects to be independent among genes. The error term $\mathbf{e_{R}}$ in the first regression follows $ \mathcal{MN}_{n_{1}\times m}(0_{n_{1}\times m}, I_{n_{1}}, \Sigma _{1} ) $, where $\Sigma _{1}$ is a $(m\times m)$ diagonal matrix with a common residual variance $\sigma _{1}^{2}$. The error term $\mathbf{e_{G}}$ in the second regression follows $ \mathcal{N}(0, \sigma _{2}^{2} I_{n_{2}} ) $. Crucially, the $m\times 1$ vector $\mathbf{\beta }$ capture the effects of gene expression on the phenotype and are our parameters of interest. We assume both $\mathbf{Y}$ and $\mathbf{Z}$ are centered so the intercepts are not included. In real data, we further adjust for commonly used covariates (e.g., age, sex and BMI) and population stratification by regressing phenotype on these confounders and use the residuals after adjustment $\tilde{\mathbf{Z}}$ in the second regression to implement the model in (1).

Viewing $\mathbf{U}$ as a matrix of latent variables, we can write out the complete-data log-likelihood and then use standard expectation-maximization (EM) algorithm to estimate the parameters of interest. For polygenic phenotype/trait that can be potentially affected by multiple genes across the genome, the genes are naturally grouped in pathways or gene sets that represent different biological processes (e.g., those pathways in KEGG pathway database [16] or gene sets from MSigDB [17]), we are particularly interested in learning what biological processes are most related to the phenotype. In addition, we are also interested in learning what specific genes within those processes are most critical in regulating the phenotype. To dissect both gene and pathway level contribution to the complex polygenic phenotype, we propose a sparse group lasso penalty [18] on $\mathbf{\beta }$ and the penalized complete-data log-likelihood is given as follows:


(2) \begin{align*}& \begin{split} & p\ell(\mathbf{Y},\mathbf{W^{(R)}}, \mathbf{W^{(G)}}, \mathbf{Z}, \mathbf{U} | \mathbf{\theta} ) = \\ & -\frac{n_{1} m} {2} \log \left(2 \pi \sigma_{1}^{2}\right) -\frac{1}{2 \sigma_{1}^{2}} \sum\limits_{i=1}^{n_{1}} (\mathbf{Y}_{i.}-\mathbf{W}^{(R)}_{i.} \mathbf{U}) (\mathbf{Y}_{i.}-\mathbf{W}^{(R)}_{i.}\mathbf{U})^{T} \\ & -\frac{n_{2} m}{2} \log \left(2 \pi \sigma_{2}^{2}\right) -\frac{1}{2 \sigma_{2}^{2}}\left\|\mathbf{Z -W}^{(G)} \mathbf{U} \beta \right\|^{2} \\ & -\frac{p}{2} \sum\limits_{j=1}^{m} \log (2 \pi \sigma_{u_{j}}^{2}) - \sum\limits_{j=1}^{m} \frac{u_{j}^{T} \mathbf{I}_{p} u_{j}}{2 \sigma_{u_{j}}^{2}} \\ & - \Bigg\{ a \lambda ||\mathbf{\beta}||_{1} + (1-a)\lambda \sum_{g=1}^{G} \sqrt{k_{g}} \|\beta^{(g)} \|_{2} \Bigg\}, \end{split}\end{align*}

where $u_{j}$ is the $j$th column of $\mathbf{U}$, $\mathbf{Y}_{i.}$, $\mathbf{W}^{(R)}_{i.}$ and $\mathbf{W}^{(G)}_{i.}$ are $i$th row of $\mathbf{Y}$, $\mathbf{W}^{(R)}$ and $\mathbf{W}^{(G)}$, $||.||_{1}$ and $||.||_{2}$ are $\ell _{1}$ and $\ell _{2}$ norms. $\mathbf{\theta }=\{ \mathbf{\beta }, \sigma _{u_{j}}^{2}, \sigma _{1}^{2}, \sigma _{2}^{2} \} $ are the parameters that need to be estimated. In this formulation, the first line on the right side corresponds to the log-likelihood in the reference data (the first regression in equation (1)), the second line corresponds to the log-likelihood in the GWAS dataset (the second regression in equation (1)), the third line corresponds to the log-likelihood of prior on $\mathbf{U}$ and the fourth line is the sparse group lasso penalty imposed on the coefficient vector $\mathbf{\beta }$. The $m$ genes are classified into $G$ pathway groups where $g=1,2,\ldots ,G$ indicates the group index and $k_{g}$ indicates the group size for the $g$th group. Scattered genes not belonging to any groups will have $k_{g}=1$. Possible overlap of genes between pathways might exist, we consider a widely used overlapping group lasso method [19] by duplicating the columns of overlapping genes from different groups in the design matrix $ \mathbf{W^{(G)} U }$ and then aggregating the coefficients corresponding to the same gene for gene-level inference. $\lambda $ is the regularization parameter, $a \in [0,1]$ indicates a convex combination of lasso (i.e. $||\mathbf{\beta }||_{1}$) and group lasso penalties (i.e. $\sum _{g=1}^{G} \sqrt{k_{g}} \|\beta ^{(g)} \|_{2} $). We use grid search with cross-validation (CV) that minimize the Mean Squared Error (MSE), as well as model selection criteria like BIC to find the optimal values of $\lambda $ and $a$. In practice, we also consider the ‘one standard error rule’ for model selection when necessary to pick a more parsimonious model within one standard error of the minimum CV error or BIC [20].

### EM algorithm for penalized likelihood

In the presence of latent variables in $\mathbf{U}$ matrix, we propose an EM algorithm to estimate the parameters that maximize the penalized likelihood function in equation (2). From the complete data penalized log-likelihood and given the block matrix structure of $\mathbf{U}$, it is easy to recognize that, given $\mathbf{Y}$, $\mathbf{W^{(R)}}$, $\mathbf{W^{(G)}}$, $\mathbf{Z}$ and $\mathbf{\theta }$, terms involving each $u_{j}$ can be written as a quadratic form for $j = 1,...,m$:


\begin{align*} &u_{j}^{T} \left(-\dfrac{1}{2\sigma_{1}^{2}} \mathbf{W^{(R)T} W^{(R)}} - \dfrac{\beta_{j}^{2}}{\sigma_{2}^{2}} \mathbf{W^{(G)T} W^{(G)}} - \dfrac{1}{2\sigma_{u_{j}}^{2}}\mathbf{I}_{p} \right) u_{j} +\\ &\left(\frac{1}{\sigma^{2}_{1}} y_{j}^{T} \mathbf{W^{(R)}} + \frac{\beta_{j}}{\sigma_{2}^{2}}\mathbf{Z^{T} W^{(G)}} \right) u_{j} + \text{constant}, \end{align*}

where $y_{j}$ is the $j$th column of $\mathbf{Y}$, $\beta _{j}$ is the corresponding coefficient for $j$th gene. Thus its posterior distribution can be written in the form of a normal distribution. In the E-step, we will take expectation of the complete data penalized log-likelihood with respect to the posterior distribution of $u_{j}$ and derive the $ Q $-function by utilizing the fact that $E(u^{T} X u )= \mu _{u}^{T} X \mu _{u} + Tr(X\Sigma _{u}) $ if $u \sim N(u|\mu _{u},\Sigma _{u})$:


(3) \begin{align*}& \begin{split} &Q(\theta|\theta_{old}) =-\frac{n_{1} m} {2} \log \left(2 \pi \sigma_{1}^{2}\right) - \sum\limits_{j=1}^{m}\frac{\mathbf{\mu_{u_{j}}}^{T}\mathbf{\mu_{u_{j}}}}{2 \sigma_{u_{j}}^{2}} \\ & -\frac{1}{2 \sigma_{1}^{2}} \sum\limits_{j=1}^{m}\sum\limits_{i=1}^{n_{1}} (\mathbf{Y}_{i.}-\mathbf{W}^{(R)}_{i.} \mathbf{\mu_{u_{j}}}) (\mathbf{Y}_{i.}-\mathbf{W}^{(R)}_{i.}\mathbf{\mu_{u_{j}}})^{T} \\ & -\frac{n_{2}m}{2} \log \left(2 \pi \sigma_{2}^{2}\right) -\frac{1}{2 \sigma_{2}^{2}}\sum\limits_{j=1}^{m}\left\|\mathbf{Z -W}^{(G)} \mathbf{\mu_{u_{j}}} \beta_{j} \right\|^{2} \\ & - \sum\limits_{j=1}^{m}\text{Tr} \left(\left( \frac{1}{2\sigma_{1}^{2}} \mathbf{W}^{(R)T} \mathbf{W^{(R)}}+ \frac{\beta_{j}^{2}}{2\sigma_{2}^{2}} \mathbf{W}^{(G)T} \mathbf{W}^{(G)} + \frac{1}{2\sigma_{u_{j}}^{2}} \mathbf{I}_{p} \right) \Sigma_{u_{j}} \right) \\ & - \Bigg\{ a \lambda ||\mathbf{\beta}||_{1} + (1-a)\lambda \sum_{g=1}^{G} \sqrt{k_{g}} \|\beta^{(g)} \|_{2} \Bigg\}, \end{split}\end{align*}

where $\Sigma _{u_{j}}= \Big( \frac{1}{\sigma _{1}^{2}} \mathbf{W}^{(R)T} \mathbf{W}^{(R)}+\frac{\beta _{j}^{2}}{\sigma _{2}^{2}} \mathbf{W}^{(G)T} \mathbf{W}^{(G)}+\frac{1}{\sigma _{u_{j}}^{2}} \mathbf{I}_{p} \Big)^{-1}$ and $\mu _{u_{j}} = \Sigma _{u_{j}} \left (\frac{1}{\sigma _{1}^{2}} \mathbf{W}^{(R)T} y_{j}+\frac{\beta _{j}}{\sigma _{2}^{2}} \mathbf{W}^{(G)T} \mathbf{Z}\right ) $.

In the M-step, we obtain the updated model parameters in $\theta $ by setting the derivative of $ Q $-function to zero. The updating equations for the parameters of interest are as follows:


\begin{align*} &\sigma_{1}^{2} =\frac{1}{n_{1}m}\left( \sum_{j=1}^{m}\left\|y_{j}- \mathbf{W^{(R)}}\mu_{u_{j}} \right\|^{2}\right), \sigma_{2}^{2} =\frac{1}{n_{2}m}\left(\left\|\mathbf{Z}-\beta_{j} \mathbf{W}^{(G)} \mu_{u_{j}}\right\|^{2}\right), \\ &\sigma_{u_{j}}^{2} =\frac{1}{p}\left({\mu_{u_{j}}}^{T} \mu_{u_{j}}+\operatorname{Tr}\left(\Sigma_{u_{j}}\right)\right), \text{ for} j=1,2,\ldots,m, \\ &\beta_{j} = \begin{cases} 0, \quad \text{if} ||s_{j}|| \leq (1-a)\lambda \sqrt{k_{g}}\ \text{or}\ ||s_{j}|| \leq a\lambda \\~\\ \left(2\mu_{u_{j}}^{T} \mathbf{W}^{(G)T} \mathbf{W}^{(G)} \mu_{u_{j}} +2\text{Tr}(\mathbf{W}^{(G)} \Sigma_{u_{j}}\mathbf{W}^{(G)T}) \right)^{-1} \times \\ s_{j} \left(1-\frac{(1-a)\lambda \sqrt{k_{g}}}{||s_{j}||} \right), \quad \text{Otherwise}, \end{cases} \\ & \text{ for}\ j=1,2,\ldots,m, \text{ where} s_{j} = 2\mathbf{Z}^{T} \mathbf{W}^{(G)} \mu_{u_{j}} -a\lambda \text{sign}(\beta_{j}). \end{align*}

The two steps are iteratively processed until convergence.

### Post-selection gene- and pathway-level inference

The EM algorithm estimates the coefficient vector $\mathbf{\beta }$ and selects the most critical genes and pathway groups for the trait with nonzero coefficients (i.e. $\beta \neq 0$) but cannot directly make inference about the effects. To avoid overfitting, we consider a data splitting scheme to make post-selection inference on the coefficients [21]. We first split the GWAS dataset into a GWAS training set and a GWAS testing set of about equal size. The GWAS training set and the reference dataset are used to explore the data and select genes with nonzero coefficients to the model. In the GWAS testing set, we will evaluate the significance of the coefficients of selected genes using a LRT. To test gene-level coefficient, we formalize the following hypothesis test: $H_{0}: {\beta _{j}} = 0, \quad \text{vs.} \quad H_{1}: {\beta _{j}} \neq 0. $ The LRT statistics is given by $ \Lambda _{j} =\ 2 \left ( \ell (\mathbf{Y},\mathbf{W^{(R)}}, \mathbf{W^{(G)}}, \mathbf{Z}, \mathbf{U} | \hat{\mathbf{\theta }} ) - \ell (\mathbf{Y},\mathbf{W^{(R)}}, \mathbf{W^{(G)}}, \mathbf{Z}, \mathbf{U} | \hat{\mathbf{\theta }}_{\beta _{j} = 0} ) \right )$, where $\hat{\theta }$ is the vector of coefficient estimates under the full model while $\hat{\mathbf{\theta }}_{\beta _{j} = 0}$ is the coefficient estimates under $H_{0}$, and $\ell (.)$ represents the likelihood function without penalty. The test statistics $\Lambda _{j}$ asymptotically follows the $\chi ^{2}$ distribution with degree of freedom of 1 under the null [22]. Likewise, we can use a similar LRT to test for pathway-level coefficient vector under the following hypothesis: $H_{0}: {\beta }^{(g)} = 0, \quad \text{vs.} \quad H_{1}: {\beta }^{(g)} \neq 0, $ where ${\beta }^{(g)}$ represents the coefficient vector for the $g$th pathway group. The pathway-level LRT statistics $ \Lambda ^{(g)} = 2 \left ( \ell (\mathbf{Y},\mathbf{W^{(R)}}, \mathbf{W^{(G)}}, \mathbf{Z}, \mathbf{U} | \hat{\mathbf{\theta }} ) - \ell (\mathbf{Y},\mathbf{W^{(R)}}, \mathbf{W^{(G)}}, \mathbf{Z}, \mathbf{U} | \hat{\mathbf{\theta }}_{\beta ^{(g)} = 0} ) \right )$, where $\hat{\theta }$ is the vector of coefficient estimates under the full model while $\hat{\mathbf{\theta }}_{\beta ^{(g)} = 0}$ is the coefficient estimates under $H_{0}$. The test statistics $\Lambda ^{(g)}$ asymptotically follows the $\chi ^{2}$ distribution with degree of freedom of $k_{g}$ under the null. We also provide a power derivation of the LRT in our method in the Supplement.

### Other related methods

Many methods have been developed to perform TWAS in the past decade (see a review of TWAS method development in the Supplement). These methods are either performed in two-stages or in joint model, use individual or summary level GWAS data, and conduct single-gene or multi-gene analysis. Table 1 summarizes the characteristics of representative TWAS methods (focusing on methods using individual level GWAS data) in each category and its comparison with our method. PrediXcan [4] is the very first two-stage univariate TWAS method. CoMM [10] is the first joint model that combines imputation and analysis stages and conducts univariate TWAS only. kTWAS [23] and later on mkTWAS [24] used kernel machine to select genetic variants but are still univariate at gene level. MV-IWAS [13] and TWAS-LQ [14] are multivariate TWAS methods but do not incorporate the pathway information nor conduct feature selection in their implementation. NeRiT [25] incorporates pathway information as network edges and performs network regression without any feature selection, thus is easily prone to overfitting. On the other hand, our method ‘TIPS’ conducts multivariate TWAS that uses pathway group information from existing pathway database and incorporates sparse group lasso penalty function to select the most critical genes and pathways that contribute to a complex trait.

**Table 1:** Main characteristics of different TWAS methods and their comparison to our TIPS method.

**Method**	**Joint/Two stage**	**Univariate/Multivariate**	**Pathway info.**	**Gene/pathway selection**	**Ref**
PrediXcan	Two-stage	Univariate	No	No	[4]
CoMM	Joint	Univariate	No	No	[10]
kTWAS	Two-stage	Univariate	No	No	[23]
mkTWAS	Two-stage	Univariate	No	No	[24]
MV-IWAS	Two-stage	Multivariate	No	No	[13]
TWAS-LQ	Two-stage	Multivariate	No	No	[14]
NeRiT	Two-stage	Multivariate	Yes	No	[25]
TIPS	Joint	Multivariate	Yes	Yes	Ours

## Application to TWAS analysis in UKB

### TWAS analysis on blood pressure

High blood pressure or hypertension is one of the leading risk factors for stroke and coronary artery disease, contributing to approximately 10 million deaths worldwide each year. Blood pressure is a complex heritable (heritability$\sim $50%) and polygenic trait potentially impacted by hundreds to thousands of variants over the whole genome based on large-scale GWAS [26]. Few TWAS have been conducted to identify the genes that contribute to blood pressure variation and understand its genetic regulatory mechanism. In this subsection, we applied our method to perform a multivariate TWAS on two traits of blood pressure: SBP and DBP to identify the genes and pathways that influence blood pressure using data from the large-scale UKB [27].

We collected the genotype, SBP, DBP and demographic data (e.g. age, sex, and BMI) from non-pregnant, family-unrelated individuals of European ancestry in UKB with at least two blood pressure measurements. Both SBP and DBP values were calculated as the mean of two nonnull blood pressure measurements using phenotype code 4080 and 4079 in UKB. After excluding subjects with missing data and those with various types of cardiovascular diseases, the complete data we analyzed include $n=16\,470$ and $17\,394$ participants for SBP and DBP, respectively. We first performed standard QC of the GWAS data and only kept the genetic variants with: minor allele frequency (MAF) $\ge 0.05$, Hardy-Weinberg equilibrium $P$-value $\ge $ 0.001, missing genotype rate $\le 0.1$. We then matched the GWAS SNP data with the genotype data in GTEx reference database (downloaded from dbGaP website, under phs000424.v9.p2) and mapped SNPs to genes in GTEx by physical proximity ($1$Mb upstream and downstream of the transcription start site, i.e. focusing on cis-eQTLs) on the reference genome. To address the lack of consensus on the physical distance used to define cis-eQTLs and minimize the impact from these different definitions on the final TWAS results, we followed from PrediXcan [4] and further performed elastic net as in PrediXcan to pre-select the most predictive SNPs for each gene among the cis-eQTLs to ensure more stable selection and reduce computational burden. The same idea holds potential when we plan to include trans-eQTLs into our model in future extensions. For the SBP and DBP traits, we used the genotype and gene expression data from the heart left ventricle tissue in GTEx (GTEx v8; RNA-seq data) to train the imputation model for the tissue’s close relationship with blood pressure. We used the pathway information from large curated pathway databases including KEGG [16], Reactome [28] and Biocarta [29] to group the genes. Pathway database like Gene Ontology are largely based on prediction so are not included here. Pathways with size smaller than 5 and large than 100 were excluded to ensure the best interpretability and biological relevance of the selected pathways. After the preparation steps, our final TWAS analysis included 2089 genes, with 848 genes grouped in 438 pathways and the remaining genes as scattered genes. To mitigate the confounding effects from other covariates, we standardized and regressed SBP and DBP phenotype on sex, age and BMI and took the residual as the adjusted SBP and DBP phenotype for TWAS analysis. The GWAS data was split into a training set and a testing set. In the training set, we ran our TIPS method to select the top genes and pathways predictive of SBP and DBP. In the testing set, we performed post-selection LRT for genes and pathways selected in the training set and obtained the $P$-values.

**Figure 2:**
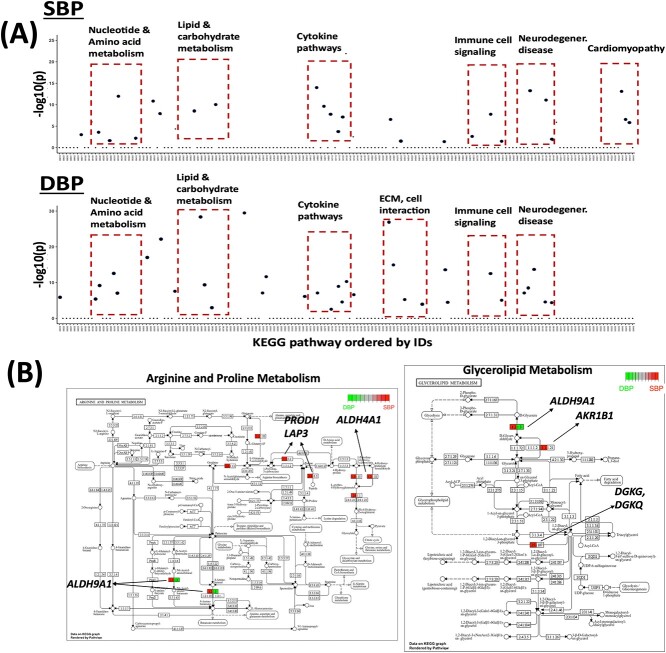
(A) Manhattan plot of -log10($P$-value) from pathway level test results of TIPS sorted by KEGG IDs (each dot represents a pathway) for SBP (upper) and DBP (lower) traits. (B) Topology plot of two significant KEGG pathways: Arginine and Proline metabolism and Glycerolipid metabolism. Selected genes in the pathway are highlighted for SBP (right) and DBP (left).

We identified 87 and 96 significant pathways for SBP and DBP, respectively, with Bonferroni adjusted $P$-value<0.05 (Table S1 and S5). Fig 2(A) and Fig S1 highlighted the significant KEGG and Reactome pathways ordered by pathway IDs, so the closer they are, the more similar their gene contents and functions. Among them, we found a few clusters of biological pathways, including nucleotide and amino acid metabolism [30, 31], lipid and carbohydrate metabolism [32, 33], cytokine signaling [34], and immune signaling [35] that have been widely reported to be related to blood pressure. In addition, we also found pathways related to neurodegenerative diseases and cellular level processes such as cell motility and cell cycle worth further investigation in future studies. Interestingly, there are a large amount of similar pathways (e.g. those related to nucleotide, amino acid and lipid metabolism, and related to cytokine pathways) identified for both SBP and DBP traits, validating the high genetic correlation between the two traits. On the other hand, pathways related to cardiomyopathy are only seen for SBP while those related to ECM and cell interaction are only seen for DBP, showing the specificity of the two traits.

We also ran CoMM, PrediXcan and MV-IWAS and applied the same cutoff to detect significant genes and performed pathway enrichment analysis on these genes for a fair comparison. For SBP, the enrichment analysis identified 8, 57, and 36 significant pathways with Benjamini–Hochberg adjusted $P$-value<0.05 for CoMM, PrediXcan, and MV-IWAS, respectively. For DBP, the enrichment analysis identified 38, 40, and 163 significant pathways for CoMM, PrediXcan, and MV-IWAS, respectively. Important pathways related to lipid and glucose metabolism had large $P$-values thus were missing from their top lists (see Table S2–4, S6–8 for a complete list and Fig. S2 for the Venn diagram). This demonstrates the power of performing multivariate TWAS and highlighted the importance of incorporating pathway group information earlier in the model to encourage pathway selection, as compared to the traditional univariate TWAS + pathway enrichment analysis that lacks unifying biological themes. Due to the lack of feature selection, the multivariate MV-IWAS method is subject to serious collinearity issues which makes it unstable resulting in very different sets of detected pathways for SBP and DBP. As a sensitivity analysis of possible false positives generated from our method, we also performed random grouping of the genes into groups of comparable sizes (repeated five times) and applied our method to each random grouped data and compared the significance of top selected pathways to our current results. As shown, top pathways based on real pathway group information from pathway database are far more significant than those with random grouping validating our current results (Fig. S3).

The pathway level comparison is not all fair for multivariate vs. univariate TWAS methods, so we also compared the different methods at gene level. For SBP, our method selected 285 significant genes while CoMM, PrediXcan, and MV-IWAS selected 140, 184, and 208 significant genes respectively with Bonferroni adjusted $P$-value<0.05; for DBP, we selected 312 genes while CoMM, PrediXcan and MV-IWAS selected 165, 215, and 356 significant genes respectively (see Fig. S4 for the Venn diagram). Multivariate TWAS methods are in general more powered than univariate TWAS methods, and the gain of power for our method mainly comes from the group-based selection that potentially aggregates signals in the same group(s). We picked two example KEGG pathways and highlighted the selected genes within the selected pathways by our method in the KEGG topology plots (Fig. 2(B)). Significant genes including aldehyde dehydrogenase (*ALDH*) genes in arginine and proline metabolism and *AKR1B1* gene in glycerolipid metabolism pathways, have been found to be related to blood pressure in previous studies [36, 37]. These findings facilitated our understanding of the genetic basis of blood pressure and potential genetic regulatory mechanism that might be related to antihypertensive drug targets.

### TWAS analysis on WM brain aging

Brain aging involves the gradual loss of structure and function of neurons and their connections, leading to cognitive decline and increased vulnerability to neurodegenerative diseases [38, 39]. Previous studies employed a machine learning algorithm and leverage multiple fractional anisotropy (FA) tract measurements obtained from diffusion tensor imaging (DTI) data to predict the WM BAG as a marker of brain aging [40, 41]. The BAG is highly heritable [42, 43], but the genetic underpinnings of the BAG are not completely understood. In this subsection, we applied our method to identify genes and pathways that influence the BAG outcome using data from UKB.

We collected the DTI data from UKB imaging visit and preprocessed the data using ENIGMA structural and DTI pipelines [44]. The FA images were aligned onto a standard-space WM skeleton and FA measurements were derived from 39 WM tracts covering various brain regions [45]. Following our previous papers [46, 47], we applied a random forest regression model to predict brain age from these 39 regional FA measures and derived our outcome of interest BAG as the difference between brain age and chronological age (see concept in Fig. S5) for $n$ = 11 299 participants. The larger BAG, the faster one’s brain ages as compared to their chronological age. The GWAS data was preprocessed as in the blood pressure example and the GTEx reference panel data for the brain cortex tissue was used to train the imputation model. As before, the curated KEGG, Reactome, and Biocarta pathway databases were used to group the genes.

**Figure 3:**
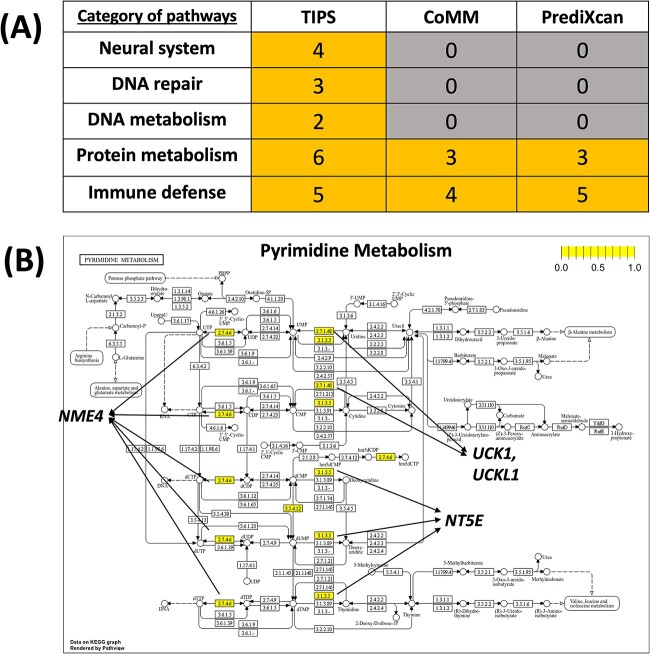
(A) Comparison of major categories of pathways detected in different methods. Number in the cells refers to the number of significant pathways within each category. (B) Topology plot of selected KEGG pathway ‘Pyrimidine metabolism’ by TIPS.

We identified 136 significant pathways for BAG with Bonferroni adjusted $P$-value<0.05 (Table S9). As a comparison, CoMM, PrediXcan, and MV-IWAS detected 35, 43, and 0 significant pathways (Table S10–S12), following the TWAS+pathway enrichment analysis procedure using the same cutoffs. [48] listed a few major categories of pathways related to the genetics of BAG including neural system, DNA repair, DNA metabolism, protein metabolism, and immune defense. Among them, only TIPS detected all the five categories of pathways (Fig. 3(A)). At gene level, we had a large overlapping proportion with the other two methods but also detected more significant genes that are otherwise missed by other methods (Fig. S6). *NME4* and *NT5E* genes elected by TIPS in the ‘Pyrimidine metabolism’ pathway (Fig. 3(B)) were previously found to be related to brain health and neurodegenerative diseases [49, 50]. These findings helped identify the critical genetic risk factors and the impacted biological pathways and functions of brain aging and improved our understanding of the aging process.

## Simulation study

### Simulation setting

In this section, we conducted various simulation studies to show the performance of our TIPS method. For benchmarking, we mainly evaluated the feature selection and statistical power performance of our method as compared to other representative TWAS methods including CoMM [10], PrediXcan-EN (PrediXcan+elastic net), PrediXcan-Lasso (PrediXcan+Lasso) [4] and the multivariate MV-IWAS method [13], the network regression based NeRiT method [25], kernel machine based kTWAS method [23], and its marginal+kernel version mkTWAS method [24].

For the reference dataset, we assumed a sample size $n_{1}=300$ and $p=900$ SNPs that are potentially regulating the expression of $m=180$ genes. We assumed each gene was independently regulated by $\bar{p}_{m}=5$ different SNPs without cross-talks and each of these SNPs is in a different locus (i.e. independent lead eQTLs). The $m=180$ genes were further classified into five pathway groups with $25$ genes in each group without overlapping, and $55$ scattered genes not belonging to any pathways. To mimic the real genotype pattern, we first defined a linkage disequilibrium (LD) matrix for each gene with fixed diagonal values equal to 1 and off-diagonal values equal to 0.5, representing the pairwise LD between SNPs within the same gene. We then followed from [51] to simulate the genotype data (i.e. the number of minor alleles for all SNPs) for each sample in the reference dataset using a latent Gaussian model with MAF$\in (0.2,0.5)$ and block-diagonal correlation matrix where each block was defined by the aforementioned LD matrix. For a complete evaluation, we followed from [23] and considered different genetic architectures: additive (2 vs 1 vs 0 minor alleles); heterogeneous (any vs none minor alleles); recessive (two alternative alleles vs others); and compensatory (one alternative allele vs others). The matrix of genetic effects was drawn as $\mathbf{U} \sim \mathcal{MN}(0, I_{p}, \text{Diag}(\sigma ^{2}_{u}) )$, assuming a common $\sigma ^{2}_{u}$ for all genes. The gene expression matrix $\mathbf{Y}$ of the reference dataset was simulated based on the first regression equation in (1), where $\sigma ^{2}_{1}$ varied depending on the scenarios (see below).

For the GWAS dataset, we assumed a sample size $n_{2}=600$, the genotype data was similarly simulated as in the reference dataset. The coefficient vector $\mathbf{\beta }$ was constructed to reflect both pathway group- and individual gene-level contribution to the trait: Group 1 that includes non-associated genes (all $\beta =0$); Group 2 that includes genes with homogeneous moderate effect (all $\beta =0.2$); Group 3 that includes genes with homogeneous weak effect (all $\beta =0.02$); and Group 4 and 5, more heterogeneous, that include genes with moderate effect, weak effect, and non-associated genes. The 55 scattered genes also included a mixture of moderate effect, weak effect, and non-associated genes. The phenotypic data was simulated based on the second regression equation in (1).

Following [10], we considered two heritability measures in simulating the data. The first cellular level heritability $\hat{h}^{2}_{C} = \frac{\bar{p}_{m}{\hat{\sigma }}^{2}_{u}}{\bar{p}_{m} {\hat{\sigma }}^{2}_{u} + {\hat{\sigma }}^{2}_{1}}$ quantifies the proportion of variance of gene expression explained by genetic variants, where $\bar{p}_{m}=5$ in our simulations. The second organism level heritability $\hat{h}^{2}_{T} = \frac{ \hat{\beta }^{T} \hat{\beta } \hat{\sigma }^{2}_{u}}{\hat{\beta }^{T} \hat{\beta } \hat{\sigma }^{2}_{u}+ \hat{\sigma }^{2}_{2}}$ quantifies the gene effect on the trait. We simulated the following three scenarios with different values of $\hat{\sigma }^{2}_{1}$, $\hat{\sigma }^{2}_{2}$, and $\hat{\sigma }^{2}_{u}$ to reflect varying cellular and organism level heritability. By adjusting heritability parameters, it demonstrates the model’s robustness in diverse biological contexts and its applicability in understanding gene–environment interactions.

1. Scenario I: $\hat{\sigma }^{2}_{1}=0.2, \hat{\sigma }^{2}_{2}=0.2, \hat{\sigma }^{2}_{u}=0.02$, $\hat{h}^{2}_{C} =0.33$ and $\hat{h}^{2}_{T} = 0.161$.
2. Scenario II: $\hat{\sigma }^{2}_{1}=0.2, \hat{\sigma }^{2}_{2}=0.2, \hat{\sigma }^{2}_{u}=0.01$; $\hat{h}^{2}_{C} =0.2$ and $\hat{h}^{2}_{T} = 0.091$.
3. Scenario III: $\hat{\sigma }^{2}_{1}=0.2, \hat{\sigma }^{2}_{2}=0.45, \hat{\sigma }^{2}_{u}=0.01$; $\hat{h}^{2}_{C} =0.2$ and $\hat{h}^{2}_{T} = 0.043$.

We also considered additional simulation scenarios when the number of genes exceeds the sample size of GWAS dataset (high-dimensional (high-D) case), when the genes are correlated and when MAF and heritability levels change.

Each simulation was conducted $ B=100 $ times. For all scenarios under investigation, we quantified the feature selection and power by plotting the Receiver Operating Characteristic (ROC) Curve (with Area Under Curve (AUC) values) and bar graphs. Five-fold CV was used to find the optimal value of regularization parameter for our method. AUCs for all univariate TWAS methods (CoMM, PrediXcan, kTWAS and mkTWAS) were calculated based on the ranking of genes by the $P$-values they provided. The true model is a multi-gene model (e.g. MV-IWAS and TIPS), so we also evaluated the predictive accuracy of the model using the MSE. MV-IWAS did not include a penalty function so cannot work in high-D scenario.

### Simulation results

**Figure 4:**
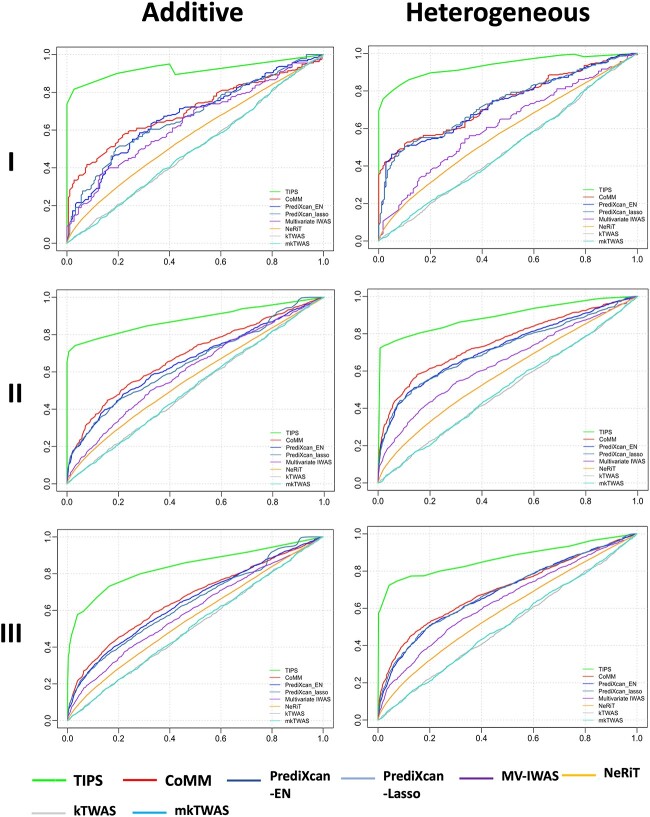
ROC curve comparison for the simulation scenarios I–III with additive or heterogeneous genetic architecture.

**Figure 5:**
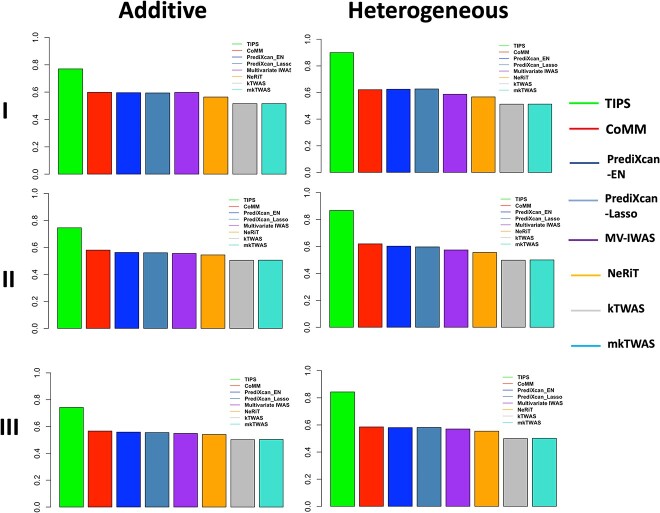
Power comparison for the simulation scenarios I–III with additive or heterogeneous genetic architecture.

Figures 4 and 5 show the ROC curve and power comparison and Table 2 shows the corresponding AUC values for all TWAS methods in different simulation scenarios. TIPS demonstrates higher AUC and power than other methods in all scenarios for different genetic architectures (additive or heterogeneous in Figs 4 and 5, recessive and compensatory in Figs S7–S8). As the heritability level drops from Scenario I to Scenario II and III, all methods have decreased AUC and power but TIPS remains the best performer. Overall, the joint models (TIPS and CoMM) that accounts for the imputation uncertainty outperform two-stage methods (PrediXcan-EN, PrediXcan-Lasso, kTWAS, and mkTWAS). The multivariate TIPS model outperforms univariate models (CoMM, PrediXcan-EN, PrediXcan-Lasso) in both feature selection and power. MV-IWAS is a multivariate model but does not include any penalty function for feature selection thus performs worse, and has higher MSE than TIPS (Table S14). NeRiT incorporates group information as network edges and runs network regression, but it is easily subject to overfitting issue thus also performs worse. The results look consistent when we consider gene–gene correlation (Fig. S9), for high-dimensional scenario (Table S15), and with other heritability levels and MAF values (Table S16 and S17).

**Table 2:** Comparison of AUC values for the simulation scenarios I-III with additive or heterogeneous genetic architecture.

	Scenario I	Scenario II	Scenario III
	Additive		
TIPS	0.953 (0.041)	0.903 (0.064)	0.833 (0.047)
CoMM	0.753 (0.042)	0.696 (0.049)	0.668 (0.034)
PrediXcan-EN	0.731 (0.041)	0.667 (0.029)	0.624 (0.035)
PrediXcan-lasso	0.728 (0.044)	0.669 (0.028)	0.621 (0.032)
MV-IWAS	0.631 (0.044)	0.610 (0.040)	0.604 (0.043)
NeRiT	0.571 (0.042)	0.541 (0.043)	0.533 (0.045)
kTWAS	0.501 (0.055)	0.504 (0.048)	0.499 (0.048)
mkTWAS	0.501 (0.046)	0.506 (0.050)	0.502 (0.049)
	Heterogeneous		
TIPS	0.966 (0.051)	0.923 (0.043)	0.845 (0.033)
CoMM	0.763 (0.043)	0.728 (0.026)	0.655 (0.039)
PrediXcan-EN	0.737 (0.032)	0.691 (0.055)	0.645 (0.039)
PrediXcan-lasso	0.743 (0.027)	0.678 (0.051)	0.647 (0.047)
MV-IWAS	0.612 (0.048)	0.591 (0.047)	0.583 (0.038)
NeRiT	0.577 (0.041)	0.554 (0.043)	0.517 (0.040)
kTWAS	0.494 (0.048)	0.491 (0.035)	0.493 (0.044)
mkTWAS	0.495 (0.048)	0.496 (0.031)	0.498 (0.031)

## Discussion

In this paper, we proposed a novel pathway-guided ‘TIPS’ method to perform multivariate TWAS analysis and identify genes and pathways that contribute to complex polygenic traits. The method is featured by its multi-gene group-based approach that examines hundreds to thousands genes simultaneously in the context of biological pathways, capturing their collective impact on the trait rather than individually. In addition, the method integrates the imputation and association stages of TWAS together to improve efficiency and employs a sparse group lasso penalty for both gene- and pathway-level feature selection. We also developed a fast EM algorithm on penalized likelihood for parameter estimation and a data splitting scheme coupled with LRT for post-selection inference at both gene and pathway levels.

In its current implementation, TIPS only considers cis-eQTLs that regulate the gene expression in reference database. Incorporating trans-eQTLs into its applications will improve the predictive performance of genetically regulated expression but is also subject to heavy computational burden due to the vast number of SNP–gene pairs that need to be tested. We have proposed one potential solution when there are too many eQTLs included by performing pre-selection of SNPs most predictive of the genes in GTEx using elastic net penalty as in PrediXcan. The applicability of such a solution for our method when incorporating all trans-eQTLs needs to be evaluated in future studies. In addition, though we considered the gene–gene correlation and grouping in the association part, the independent gene regulation assumption in the imputation part could be strong in real application. For example, it is not rare to see common genetic regulators and regulatory mechanisms of different genes in proximity or functionally related. Future extension could consider such complex multi-SNPs-to-multi-genes regulatory relationship.

The current method, tailored for individual-level GWAS data and continuous phenotype, holds potential for adaptation to accommodate GWAS summary statistics and mixed types of phenotype, broadening its applicability as in other TWAS methods [6, 52]. In addition, the current TWAS method is only trained in one tissue and performs single population analysis. Future works can be focused on extending the current TWAS method towards a joint multi-tissue model [7] and joint multi-ancestry analysis [53]. As a wide variety of phenotypes are being collected nowadays, testing multiple phenotypes simultaneously can be more powerful than testing each phenotype individually by considering the correlation across phenotypes. The current TIPS method could be readily extended to address multivariate phenotype, allowing for the investigation of a number of complex traits all together. This will likely lead to a high-dimension-to-high-dimensional problem (a large number of genes to a large number of phenotypes) that may require special care in modeling for feature screening and selection [54, 55].

There are a few existing online TWAS resources of disease susceptibility genes such as TWAS-hub [56] and webTWAS [57], our proposed TIPS method, on the other hand, is the first method that incorporates curated pathway database in multivariate TWAS model and provides pathway-level information associated with a disease or trait. It could potentially be a valuable tool offering more reliable and interpretable results for TWAS analyses. An efficient R package called ‘TIPS’ was developed (https://github.com/nwang123/TIPS) to implement the method.

**Key Points**

- Existing TWAS methods are mainly univariate by nature with little unifying biological themes, we proposed a novel pathway-guided joint model for multivariate TWAS analysis to identify both genes and pathways associated with complex polygenic traits by employing a sparse group lasso penalty and developed an efficient EM algorithm to estimate parameters for the penalized likelihood.
- Our TIPS model is the first TWAS method that simultaneously considers gene–gene correlation, gene sets with similar function as well as imputation uncertainty in TWAS to identify critical biological processes underlying a complex polygenic trait. Our multi-gene group-based approach examines hundreds to thousands of genes together in the context of biological pathways capturing their collective impact on a trait and has the great potential to provide deeper insights into the functional foundations and molecular mechanism underlying a complex trait.
- We applied our method to three different complex traits, SBP, DBP, and BAG, from UK Biobank and identified critical pathways and genes related to lipid and glucose metabolism for blood pressure as well as neural system, DNA repair, and DNA metabolism related pathways for BAG, while traditional univariate TWAS + pathway enrichment analysis approach failed to detect these important pathways. Comprehensive simulations that mimic real TWAS settings also showed the advantage of our method in feature selection, power, and prediction as compared to existing representative TWAS methods.
- With the flexible joint modeling framework, our method is readily extensible to accommodate more complex TWAS analyses with mixed types of phenotypes, multiple tissues, and ancestries.

## Supplementary Material

TIPS_R1_supp_bbae587

TIPS_R1_supp_table_bbae587
