# Supplementary material for: TIPS: a novel pathway-guided joint model for transcriptome-wide association studies
Source: Brief Bioinform. 2024 Nov 16;25(6):bbae587. doi: 10.1093/bib/bbae587 (PMC11568880; doi:10.1093/bib/bbae587)
Supplement: TIPS_R1_supp_bbae587 [file tips_r1_supp_bbae587.pdf]

Supplementary materials for “TIPS: a novel pathway-guided joint model for transcriptome-wide association studies”

Neng Wang, Zhenyao Ye and Tianzhou Ma

1 Review of TWAS method development

A typical TWAS integrates two sources of data: the genotype and gene expression data from reference panel like GTEx (Lonsdale, et al., 2013), and the GWAS (individual or summary level) data for a specific trait/phenotype. Traditional TWAS methods such as PrediXcan (Gamazon, et al., 2015) is implemented in two stages: in the first stage, the genetically regulated components of gene expression for the GWAS cohort is imputed using the genotype data and the weights trained from reference panel such as GTEx which has matched genotype and gene expression data; in the second stage, an association analysis is performed between imputed expression of each gene and the trait of interest in the GWAS cohort. Since then, a number of two-stage TWAS methods that use different statistical learning and feature selection methods in imputation stage (Gusev, et al., 2016; Zeng and Zhou, 2017), leverage information from multiple tissues to train imputation model (Barbeira, et al., 2019; Hu, et al., 2019; Shi, et al., 2020), adopting summary-level GWAS data (Barbeira, et al., 2018; Barbeira, et al., 2019; Yang, et al., 2020) have been developed to improve the performance of TWAS methods. One may refer to (Mai, et al., 2023; Xie, et al., 2021; Zhu and Zhou, 2020) for a complete list of these methods.

One major drawback of the two-stage TWAS methods is that they do not account for the uncertainty in the imputation process, leading to potential loss in power. Yang et al. (2019) proposed the first likelihood-based joint model “CoMM” that jointly models the imputation and association stages by utilizing a collaborative mixed model (Yang, et al., 2019). The joint modeling approach enhances the power and precision of genetic association studies, providing deeper insights into gene-trait correlations. Despite its merits, CoMM still conducts univariate TWAS where the association analysis is performed for each gene separately. The list of individual genes identified may contain little unifying biological theme so the underlying biological mechanisms remain largely elusive. In addition, the complex correlation between genes makes it difficult to identify the most critical and potentially causal genes of a polygenic trait.

Multivariate TWAS methods have recently emerged but their development is still at an early stage (He, et al., 2022). Mancuso et al. (2019) proposed a fine mapping method for TWAS that simultaneously models multiple genes but is restricted to specific risk regions (Mancuso, et al., 2019). In addition, their correlation structure is mainly induced by linkage disequilibrium but carries little information about the common cellular or molecular functions of the genes interacting with each other. Knutson et al. (2020) and Lin et al. (2022) proposed multivariate TWAS methods that account for the additive effects of multiple genes, or of a gene's linear and nonlinear effects based on Mendelian Randomization framework but failed to introduce any feature selection methods to select the genes or effects (Knutson, et al., 2020; Lin, et al., 2022).

Other multivariate TWAS methods are mainly designed for integrating multiple tissues in the imputation stage of TWAS (Barbeira, et al., 2019; Hu, et al., 2019).
Lastly, all these multivariate TWAS methods are strictly two-stage only. This has motivated us to develop a novel joint multi-gene model that includes the biological pathway group information (e.g. those from Kyoto Encyclopedia of Genes and Genomes (KEGG) database (Kanehisa, et al., 2016) that includes a collection of genes with common function or biological theme) for multivariate TWAS analysis and incorporates a sparse group lasso penalty to select both genes and pathways that contribute to complex polygenic traits.

2 Supplementary results

Supplementary Figures:

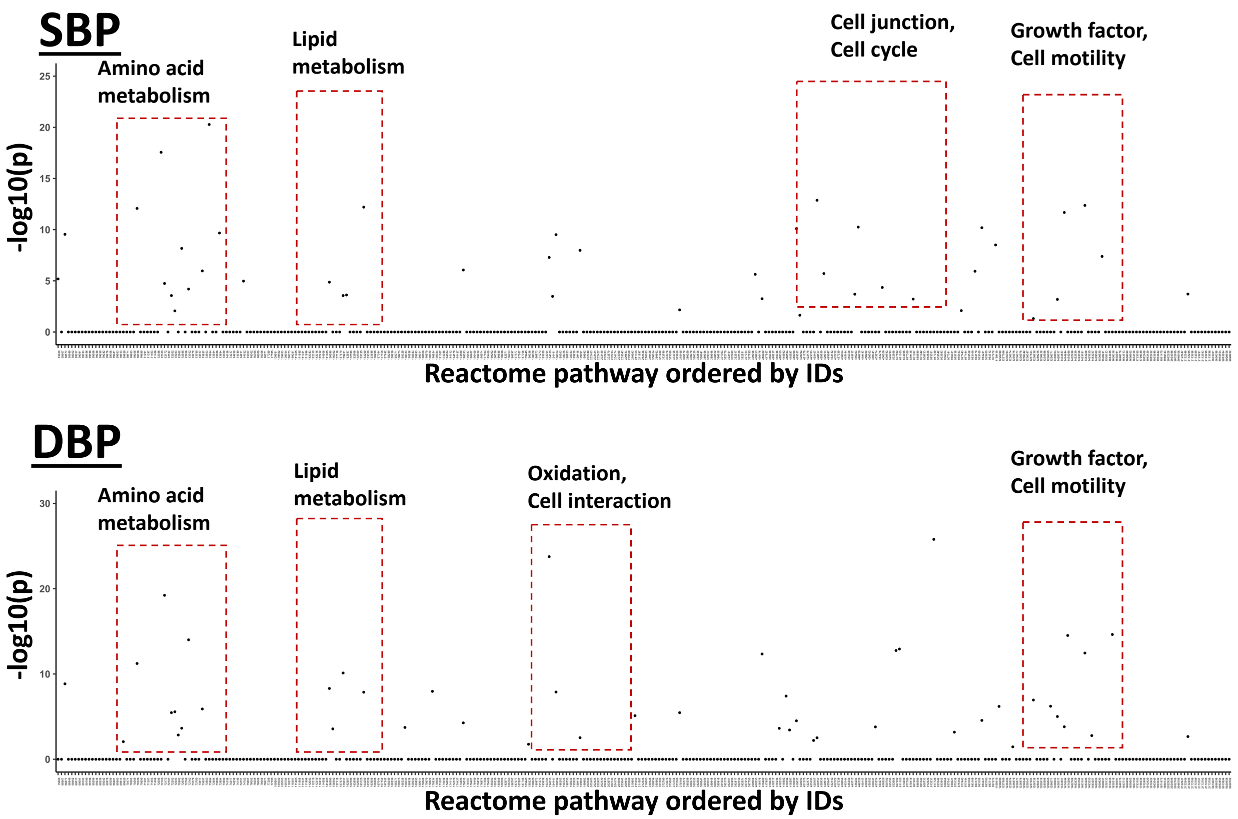

Fig S1. Manhattan plot of -log10(p-value) from pathway level test results of TIPS for SBP and DBP sorted by Reactome pathway IDs

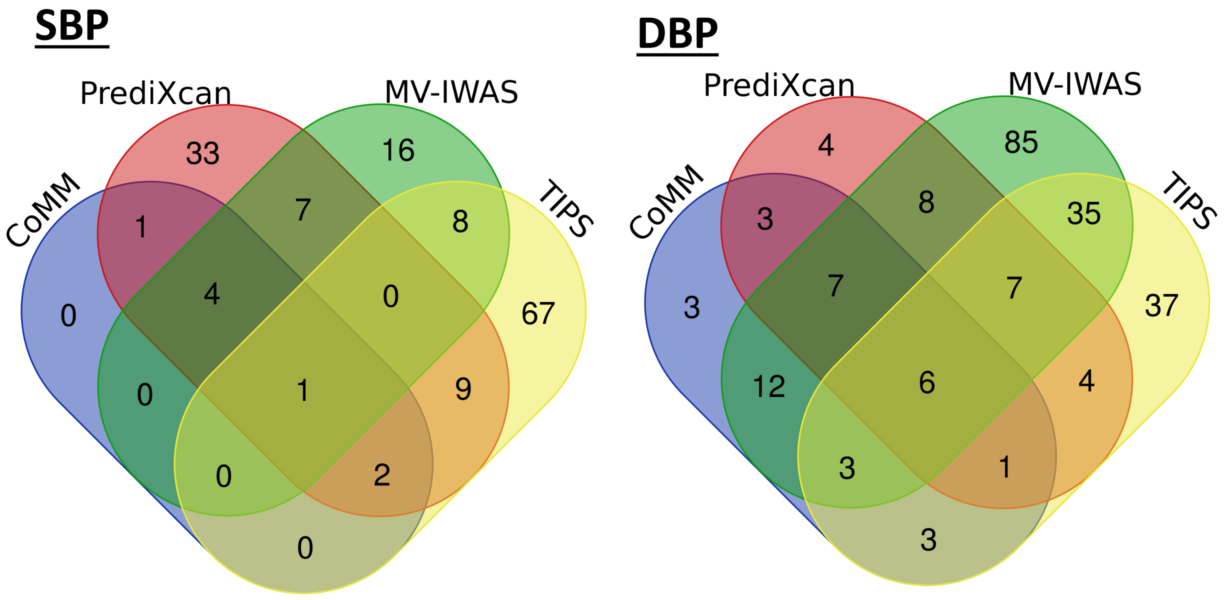

Fig S2. Venn diagram comparing the number of significant pathways identified by TIPS (from likelihood ratio test) and other TWAS methods (from pathway enrichment analysis) for the SBP and DBP example.

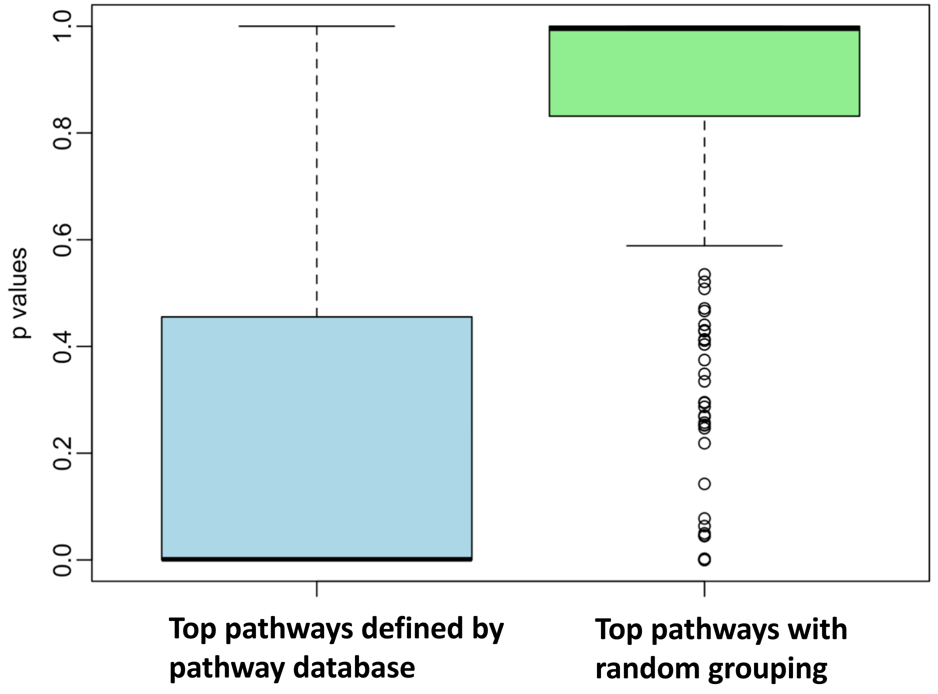

Fig S3. TIPS p-value comparison of top pathways defined by pathway database vs top pathways with random grouping (repeat for B=5 times) as a sensitivity analysis.

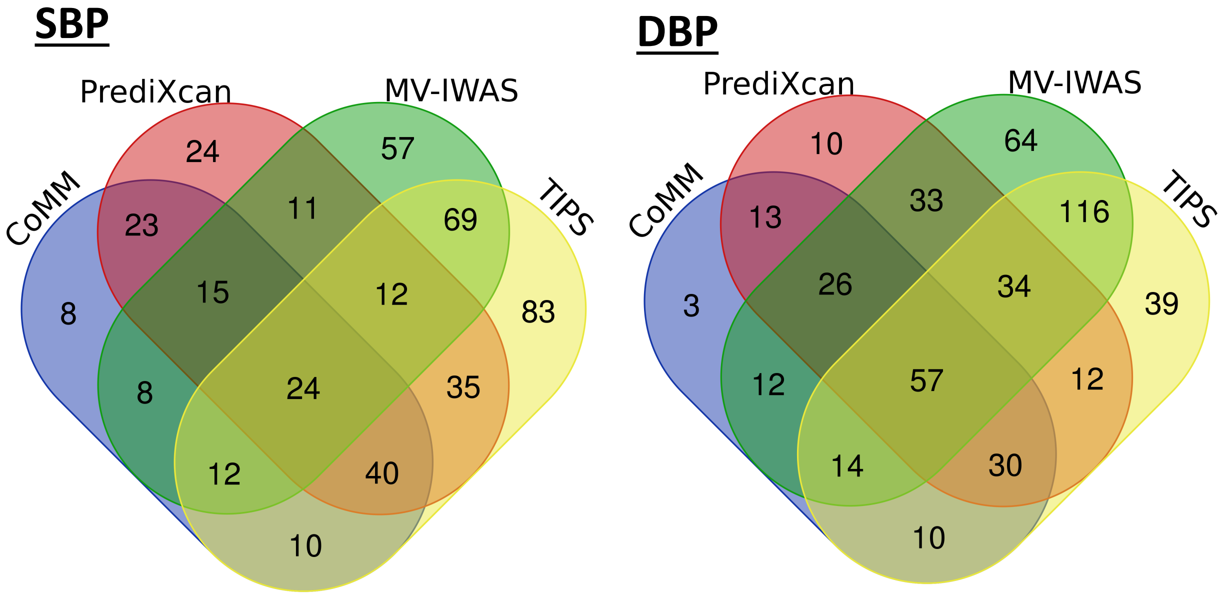

Fig S4. Venn diagram comparing the number of significant genes identified by TIPS (from likelihood ratio test) and other TWAS methods for the SBP and DBP example.

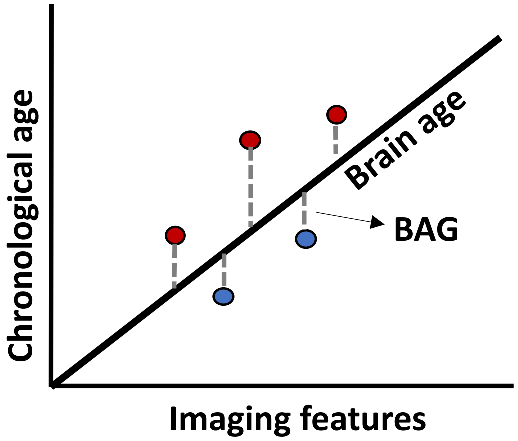

Fig S5. Concept of brain age and brain age gap (BAG).

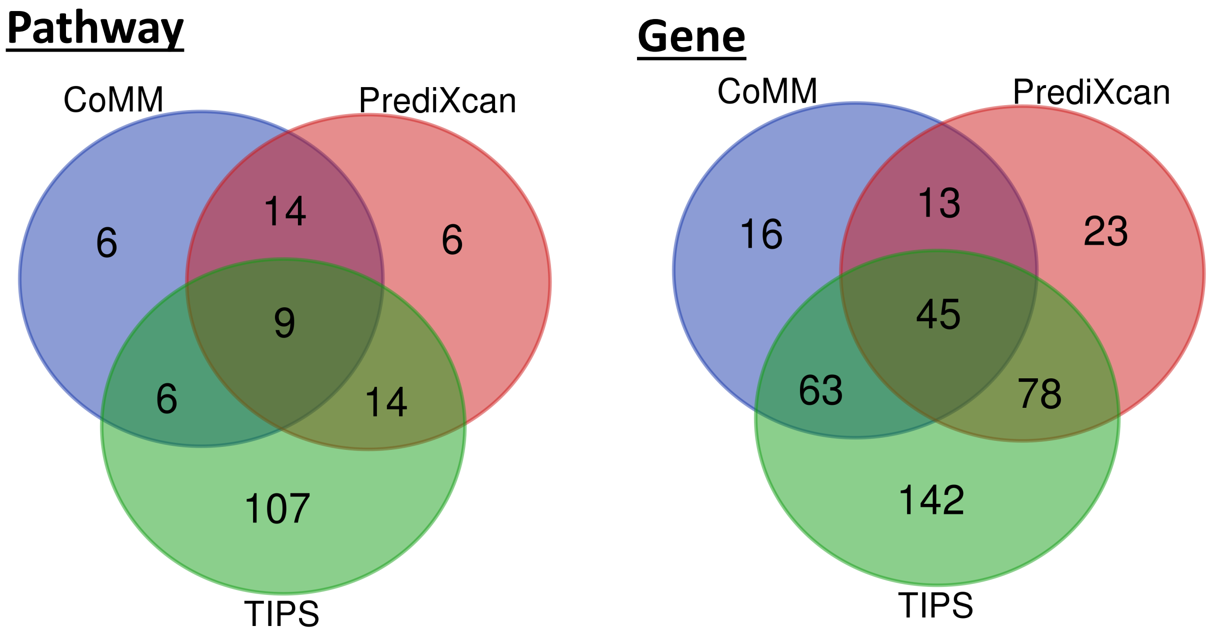

Fig S6. Venn diagram comparing the number of significant pathways (left) and genes (right) identified by TIPS and other TWAS methods for the BAG example.

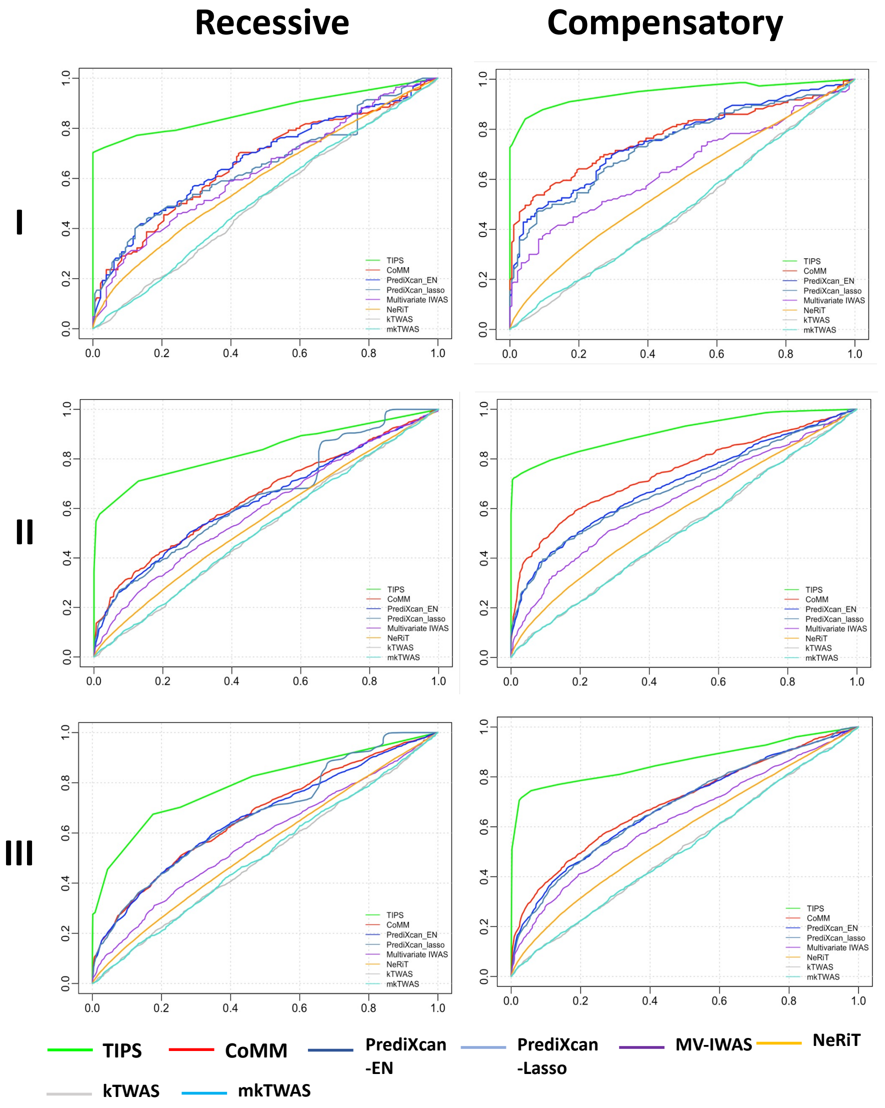

Fig S7. ROC curve comparison for the simulation scenarios I-III with Recessive or Compensatory genetic architecture.

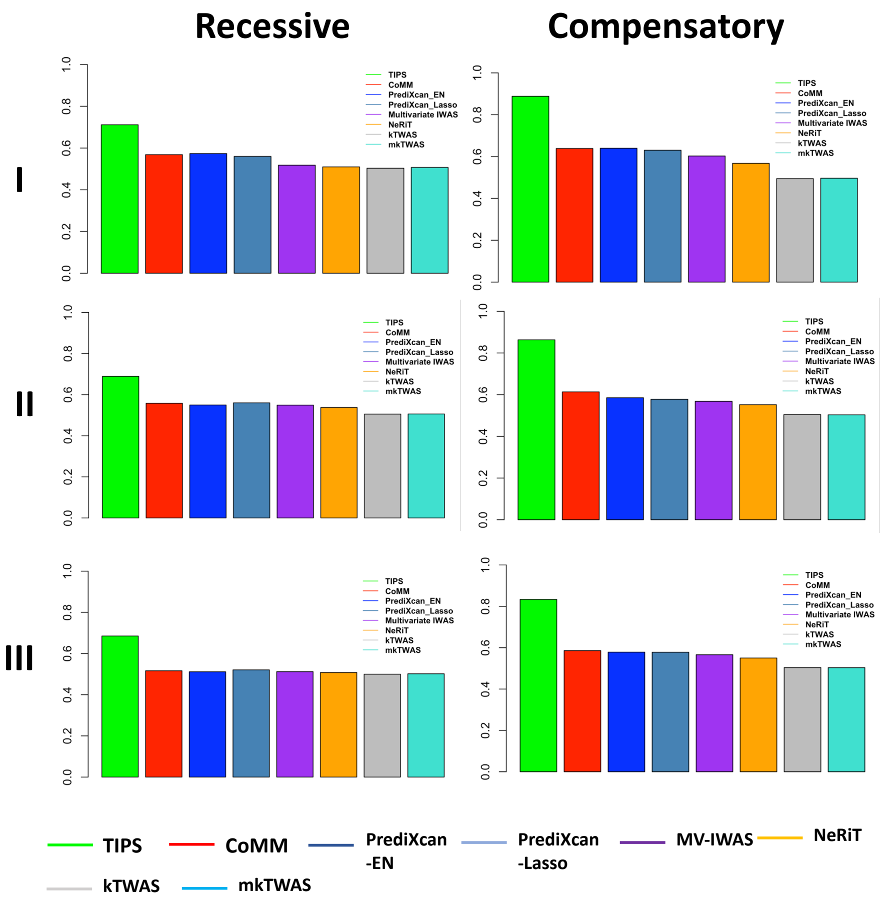

Fig S8. Power comparison for the simulation scenarios I-III with Recessive or Compensatory genetic architecture.

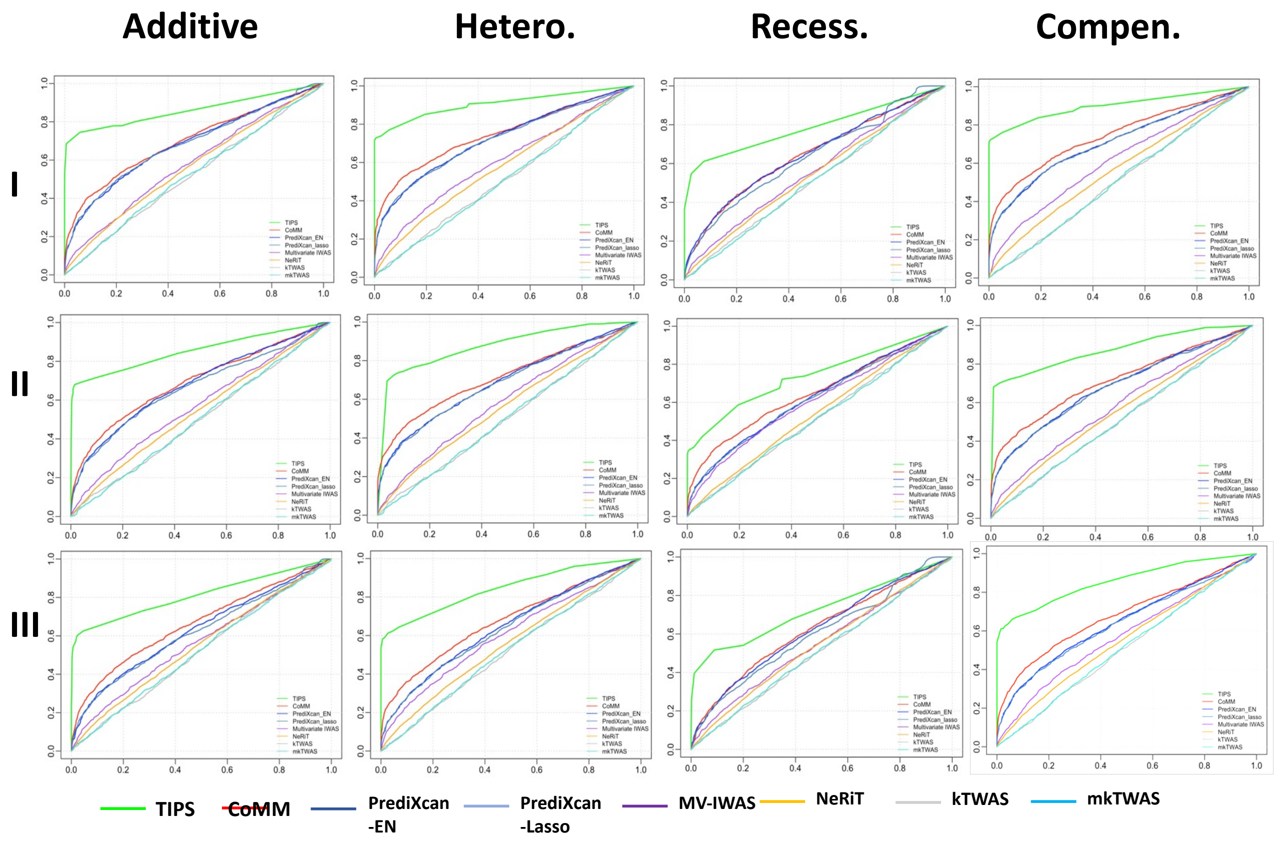

Fig S9. Additional simulation results with gene-gene correlation=0.1, ROC curve comparison for simulation scenarios I-III with Additive, Heterogenous Recessive or Compensatory genetic architecture.

Supplementary Tables:

Table S1. List of significant pathways (Bonferroni corrected p-value<0.05) identified by TIPS for SBP
Table S2. Summary of pathway enrichment analysis results using the significant genes identified by CoMM for SBP
Table S3. Summary of pathway enrichment analysis results using the significant genes identified by PrediXcan for SBP
Table S4. Summary of pathway enrichment analysis results using the significant genes identified by MV-IWAS for SBP
Table S5. List of significant pathways (Bonferroni corrected p-value<0.05) identified by TIPS for DBP
Table S6. Summary of pathway enrichment analysis results using the significant genes identified by CoMM for DBP
Table S7. Summary of pathway enrichment analysis results using the significant genes identified by PrediXcan for DBP
Table S8. Summary of pathway enrichment analysis results using the significant genes identified by MV-IWAS for DBP
Table S9. List of significant pathways (Bonferroni corrected p-value<0.05) identified by TIPS for BAG
Table S10. Summary of pathway enrichment analysis results using the significant genes identified by CoMM for BAG
Table S11. Summary of pathway enrichment analysis results using the significant genes identified by PrediXcan for BAG
Table S12. Summary of pathway enrichment analysis results using the significant genes identified by MV-IWAS for BAG (no significant pathways identified)

Table S13. Number of cis-SNPs left by considering different distances from TSS, before and after pre-selection by elastic net as in PrediXcan in GTEx data from heart tissue.

Distance from TSS	***250kb***	***500kb***	***1Mb***
Number of cis-SNPs	358602	400165	412319
Number of cis-SNPs after pre-selection by elastic net as in PrediXcan	8551	9076	9089

Table S14. MSE values for simulation I-III (low-D) for Additive genetic architecture. As the true model is a multi-gene model, we calculate the MSE only for multivariate method.

	**MSE**		
	Scenario I	Scenario II	Scenario III
TIPS	0.419 (0.411)	0.268 (0.197)	0.452 (0.468)
MV-IWAS	0.674 (0.221)	0.451 (0.185)	0.693 (0.259)

Table S15. Additional simulation results with high-dimension (high-D) scenario (n1=200, n2=300, m=400 genes mapped to 5 pathway groups, p=2000 SNPs) for Additive genetic architecture. MV-IWAS did not include a penalty function so cannot work in high-D scenario.

	**AUC**
TIPS	0.929 (0.052)
CoMM	0.652 (0.042)
PrediXcan-EN	0.621 (0.043)
PrediXcan-Lasso	0.618 (0.042)

Table S16. Additional simulation results with MAF=0.5 and varying heritability and dimensionality: Scenario IV. $\hat{h}_c^2 = 0.7$, $\hat{h}_T^2 = 0.028$; Scenario V. $\hat{h}_c^2 = 0.2$, $\hat{h}_T^2 = 0.008$; Scenario VI. $\hat{h}_c^2 = 0.2$, $\hat{h}_T^2 = 0.004$; high-D scenario. $\hat{h}_c^2 = 0.7$, $\hat{h}_T^2 = 0.028$.

	AUC			
	low-D			high-D
	Scenario IV	Scenario V	Scenario VI	
TIPS	0.925(0.045)	0.814(0.092)	0.780(0.070)	0.830(0.011)
CoMM	0.769(0.043)	0.628(0.067)	0.574(0.056)	0.555(0.032)
PredXican-EN	0.723(0.032)	0.544(0.048)	0.498(0.047)	0.545(0.035)
PredXican-Lasso	0.723(0.032)	0.559(0.047)	0.505(0.038)	0.542(0.035)

Table S17. Additional simulation results with MAF=0.5 and varying heritability and dimensionality: Scenario VII. $\hat{h}_c^2 = 0.7$, $\hat{h}_T^2 = 0.008$; Scenario VIII. $\hat{h}_c^2 = 0.2$, $\hat{h}_T^2 = 0.008$; high-D scenario. $\hat{h}_c^2 = 0.2$, $\hat{h}_T^2 = 0.008$.

	AUC		
	low-D		high-D
	Scenario VII	Scenario VIII	
TIPS	0.804(0.059)	0.805(0.052)	0.827(0.025)
CoMM	0.624(0.052)	0.622(0.064)	0.552(0.037)
PredXican-EN	0.554(0.043)	0.541(0.044)	0.541(0.042)
PredXican-Lasso	0.553(0.042)	0.533(0.038)	0.538(0.046)

References:

Barbeira, A.N., *et al.* Exploring the phenotypic consequences of tissue specific gene expression variation inferred from GWAS summary statistics. *Nat Commun* 2018;9(1):1825.

Barbeira, A.N., *et al.* Integrating predicted transcriptome from multiple tissues improves association detection. *PLoS genetics* 2019;15(1):e1007889.

Gamazon, E.R., *et al.* A gene-based association method for mapping traits using reference transcriptome data. *Nat Genet* 2015;47(9):1091-1098.

Gusev, A., *et al.* Integrative approaches for large-scale transcriptome-wide association studies. *Nat Genet* 2016;48(3):245-252.

He, R., *et al.* Statistical power of transcriptome-wide association studies. *Genetic epidemiology* 2022;46(8):572-588.

Hu, Y., *et al.* A statistical framework for cross-tissue transcriptome-wide association analysis. *Nat Genet* 2019;51(3):568-576.

Kanehisa, M., *et al.* KEGG as a reference resource for gene and protein annotation. *Nucleic acids research* 2016;44(D1):D457-D462.

Knutson, K.A., Deng, Y. and Pan, W. Implicating causal brain imaging endophenotypes in Alzheimer's disease using multivariable IWAS and GWAS summary data. *NeuroImage* 2020;223:117347.

Lin, Z., *et al.* Accounting for nonlinear effects of gene expression identifies additional associated genes in transcriptome-wide association studies. *Human molecular genetics* 2022;31(14):2462-2470.

Lonsdale, J., *et al.* The genotype-tissue expression (GTEx) project. *Nature genetics* 2013;45(6):580-585.

Mai, J., *et al.* Transcriptome-wide association studies: recent advances in methods, applications and available databases. *Communications Biology* 2023;6(1):899.

Mancuso, N., *et al.* Probabilistic fine-mapping of transcriptome-wide association studies. *Nature genetics* 2019;51(4):675-682.

Shi, X., *et al.* A tissue-specific collaborative mixed model for jointly analyzing multiple tissues in transcriptome-wide association studies. *Nucleic acids research* 2020;48(19):e109-e109.

Xie, Y., *et al.* Transcriptome wide association studies: General framework and methods. *Quantitative Biology* 2021;9(2):141-150.

Yang, C., *et al.* CoMM: a collaborative mixed model to dissecting genetic contributions to complex traits by leveraging regulatory information. *Bioinformatics* 2019;35(10):1644-1652.

Yang, Y., *et al.* CoMM-S2: a collaborative mixed model using summary statistics in transcriptome-wide association studies. *Bioinformatics* 2020;36(7):2009-2016.

Zeng, P. and Zhou, X. Non-parametric genetic prediction of complex traits with latent Dirichlet process regression models. *Nature communications* 2017;8(1):456.

Zhu, H. and Zhou, X. Transcriptome-wide association studies: a view from Mendelian randomization. *Quantitative Biology* 2020:1-15.

Supplementary Proof:

3 Derivation of the power for the post-selection likelihood ratio test in TIPS

Derivation of the power for the post-selection likelihood ratio test in TIPS

1 Post-selection likelihood ratio test (LRT)

The post-selection likelihood ratio test (LRT) statistic for our TIPS method is defined in the main text, at gene level:

$$\Lambda_j = 2\left(\ell(\mathbf{Y}, \mathbf{W}^{(\mathbf{R})}, \mathbf{W}^{(\mathbf{G})}, \mathbf{Z}, \mathbf{U}|\hat{\theta}) - \ell(\mathbf{Y}, \mathbf{W}^{(\mathbf{R})}, \mathbf{W}^{(\mathbf{G})}, \mathbf{Z}, \mathbf{U}|\hat{\theta}_{\beta_j=0})\right) \tag{1}$$

And at pathway level:

$$\Lambda^{(g)} = 2\left(\ell(\mathbf{Y}, \mathbf{W}^{(\mathbf{R})}, \mathbf{W}^{(\mathbf{G})}, \mathbf{Z}, \mathbf{U}|\hat{\theta}) - \ell(\mathbf{Y}, \mathbf{W}^{(\mathbf{R})}, \mathbf{W}^{(\mathbf{G})}, \mathbf{Z}, \mathbf{U}|\hat{\theta}_{\beta^{(g)}=0})\right) \tag{2}$$

Without loss of generality, we can simplify the above formulas as:

$$\Lambda = -2\left(\ell(\hat{\theta}_{-\beta}, \beta = \mathbf{0}) - \ell(\hat{\theta}_{-\beta}, \beta = \hat{\beta})\right), \tag{3}$$

where β may refer to coefficient for jth gene β_j or a coefficient vector for gth pathway $\beta^{(g)}$, $\hat{\theta}$ and $\hat{\beta}$ are the corresponding MLE estimates, $\hat{\theta}_{-\beta}$ is the estimate not including β. $\ell(\hat{\theta}_{-\beta}, \beta = \mathbf{0})$ is the likelihood under the null hypothesis H_0 and $\ell(\hat{\theta}_{-\beta}, \beta = \hat{\beta})$ is the likelihood under the alternative hypothesis H_a.

Under H_0, Λ follows a chi-square distribution with p degrees of freedom:

$$\Lambda \sim \chi^2_p, \tag{4}$$

where $p = 1$ for gene-level inference and $p = k_g$ for pathway-level inference.

Under H_a, Λ follows a noncentral chi-square distribution with p degrees of freedom and noncentrality parameter λ:

$$\Lambda \sim \chi^2_p(\eta) \tag{5}$$

To derive the power for our LRT test, we will need to derive the expression of η, we first derive the form of LRT statistics in our context.

2 Derivation of the LRT statistic

The LRT statistic can be further expanded as:

$$
\begin{aligned}
\Lambda &= -2\left(\ell(\hat{\theta}_{-\beta}, \beta = \mathbf{0}) - \ell(\hat{\theta}_{-\beta}, \beta = \hat{\beta})\right) \\
&= \frac{1}{\sigma_2^2}\left(\mathbf{Z}^\top\mathbf{Z} - (\mathbf{Z} - (\mathbf{W^{(G)}U})_\beta\hat{\beta})^\top(\mathbf{Z} - (\mathbf{W^{(G)}U})_\beta\hat{\beta})\right) \\
&= \frac{1}{\sigma_2^2}\left(2\mathbf{Z}^\top(\mathbf{W^{(G)}U})_\beta\hat{\beta} - \hat{\beta}^\top(\mathbf{W^{(G)}U})_\beta^\top(\mathbf{W^{(G)}U})_\beta\hat{\beta}\right),
\end{aligned}
\tag{6}
$$

where $(\mathbf{W^{(G)}U})_\beta$ refers to the column(s) of the design matrix $(\mathbf{W^{(G)}U})$ that correspond to the coefficient β.

Denote by $\mathbf{V} = (\mathbf{W^{(G)}U})_\beta$ for simplicity, we know the OLS estimates of β is: $\hat{\boldsymbol{\beta}} = (\mathbf{V}^\top\mathbf{V})^{-1}\mathbf{V}^\top\mathbf{Z}$, so we plug in and will have:

$$
\begin{aligned}
\Lambda &= \frac{1}{\sigma_2^2}\left(2\mathbf{Z}^\top\mathbf{V}(\mathbf{V}^\top\mathbf{V})^{-1}\mathbf{V}^\top\mathbf{Z} - \mathbf{Z}^\top\mathbf{V}(\mathbf{V}^\top\mathbf{V})^{-1}\mathbf{V}^\top\mathbf{V}(\mathbf{V}^\top\mathbf{V})^{-1}\mathbf{V}^\top\mathbf{Z}\right) \\
&= \frac{1}{\sigma_2^2}\left(\mathbf{Z}^\top\mathbf{V}(\mathbf{V}^\top\mathbf{V})^{-1}\mathbf{V}^\top\mathbf{Z}\right) \\
&= \frac{1}{\sigma_2^2}\mathbf{Z}^\top\mathbf{P}\mathbf{Z},
\end{aligned}
$$

where $\mathbf{P} = \mathbf{V}(\mathbf{V}^\top\mathbf{V})^{-1}\mathbf{V}^\top$ is the projection matrix onto the column space of $\mathbf{V}$.

3 Deriving the noncentrality parameter η

Under null H_0, $\Lambda \sim \chi_p^2$ so $\mathbb{E}[\Lambda \mid H_0] = p$.

As $\mathbf{Z} = \mathbf{W^{(G)}U}\beta + \boldsymbol{\varepsilon}$, denote by $\mathbf{W^{(G)}U} = \mathbf{X}$ and $\boldsymbol{\varepsilon} = \mathbf{e_G}$, we have:

$$
\begin{aligned}
\Lambda &= \frac{1}{\sigma_2^2}(\mathbf{X}\beta + \boldsymbol{\varepsilon})^\top\mathbf{P}(\mathbf{X}\beta + \boldsymbol{\varepsilon}) \\
&= \frac{1}{\sigma_2^2}\left(\beta^\top\mathbf{X}^\top\mathbf{P}\mathbf{X}\beta + 2\boldsymbol{\varepsilon}^\top\mathbf{P}\mathbf{X}\beta + \boldsymbol{\varepsilon}^\top\mathbf{P}\boldsymbol{\varepsilon}\right)
\end{aligned}
\tag{7}
$$

Under the alternative H_a, we have:

$$
\mathbb{E}[\Lambda \mid H_a] = \frac{1}{\sigma_2^2}\left(\boldsymbol{\beta}^\top\mathbf{X}^\top\mathbf{P}\mathbf{X}\boldsymbol{\beta} + 2\mathbb{E}[\boldsymbol{\varepsilon}^\top\mathbf{P}\mathbf{X}\boldsymbol{\beta}] + \mathbb{E}[\boldsymbol{\varepsilon}^\top\mathbf{P}\boldsymbol{\varepsilon}]\right) \tag{8}
$$

Since $\mathbb{E}[\boldsymbol{\varepsilon}] = \mathbf{0}$:

- $\mathbb{E}[\boldsymbol{\varepsilon}^\top\mathbf{P}\mathbf{X}\boldsymbol{\beta}] = \boldsymbol{\beta}^\top\mathbf{X}^\top\mathbf{P}\mathbb{E}[\boldsymbol{\varepsilon}] = 0$
- $\mathbb{E}[\boldsymbol{\varepsilon}^\top\mathbf{P}\boldsymbol{\varepsilon}] = \sigma_2^2\,\mathrm{tr}(\mathbf{P}) = \sigma_2^2 p$ since $\mathbf{P}$ is idempotent and symmetric with rank p

Therefore:

$$
\begin{aligned}
\mathbb{E}[\Lambda \mid H_a] &= \frac{1}{\sigma_2^2}\left(\boldsymbol{\beta}^\top\mathbf{X}^\top\mathbf{P}\mathbf{X}\boldsymbol{\beta} + 0 + \sigma^2 p\right) \\
&= \frac{1}{\sigma^2}\boldsymbol{\beta}^\top\mathbf{X}^\top\mathbf{P}\mathbf{X}\boldsymbol{\beta} + p
\end{aligned}
\tag{9}
$$

The noncentrality parameter η is defined as:

$$\begin{aligned}\eta &= \mathbb{E}[\Lambda \mid H_a] - \mathbb{E}[\Lambda \mid H_0] \\ &= \left(\frac{1}{\sigma_2^2}\boldsymbol{\beta}^\top \mathbf{X}^\top \mathbf{P}\mathbf{X}\boldsymbol{\beta} + p\right) - p \\ &= \frac{1}{\sigma_2^2}\boldsymbol{\beta}^\top \mathbf{X}^\top \mathbf{P}\mathbf{X}\boldsymbol{\beta}\end{aligned}$$

4 Derivation of the power

The power of the LRT is the probability that the test statistic exceeds the critical value under H_a:

$$\text{Power} = P\left(\Lambda > \chi^2_{p,\alpha} \mid H_a\right), \tag{10}$$

where $\chi^2_{p,\alpha}$ is the critical value from the chi-square distribution with p degrees of freedom at significance level α.

Using the cumulative distribution function (CDF) of the noncentral chi-square distribution:

$$\text{Power} = 1 - F_{\chi^2_p(\eta)}\left(\chi^2_{p,\alpha}\right) \tag{11}$$

where $F_{\chi^2_p(\eta)}$ is the CDF of the noncentral chi-square distribution, with $\eta = \frac{1}{\sigma_2^2}\boldsymbol{\beta}^\top \mathbf{X}^\top \mathbf{P}\mathbf{X}\boldsymbol{\beta}$.
